# Supplementary material for: Transcriptomics of Besnoitia besnoiti-Infected Fibroblasts Reveals Hallmarks of Early Fibrosis and Cancer Progression
Source: Microorganisms. 2024 Mar 15;12(3):586. doi: 10.3390/microorganisms12030586 (PMC10975890; doi:10.3390/microorganisms12030586)
Supplement: Supplementary file 1 [file microorganisms-12-00586-s001.zip › Supplementary Figure S2.pdf]

Supplementary Figure S2: Scatter-plot graphs of *VEGFA* relative mRNA expression levels derived from quantitative real-time PCR (RT–qPCR) analysis in human foreskin fibroblasts (HFFs) infected with *B. besnoiti* tachyzoites at 12h p.i. and treated with ERK inhibitor FR180204.

Graphical comparison of means + SD of Fold Change (2^-(ΔΔCt)) between non-infected and *Besnoitia besnoiti* infected HFFs with 0.1 µM (A), 10 µM (B) and 100 µM (C) MAPKs inhibitor. Statistical analysis was performed by Tukey's multiple comparison test. * = *p*-value <0.05; ** = *p*-value <0.01; ** = *p*-value <0.001

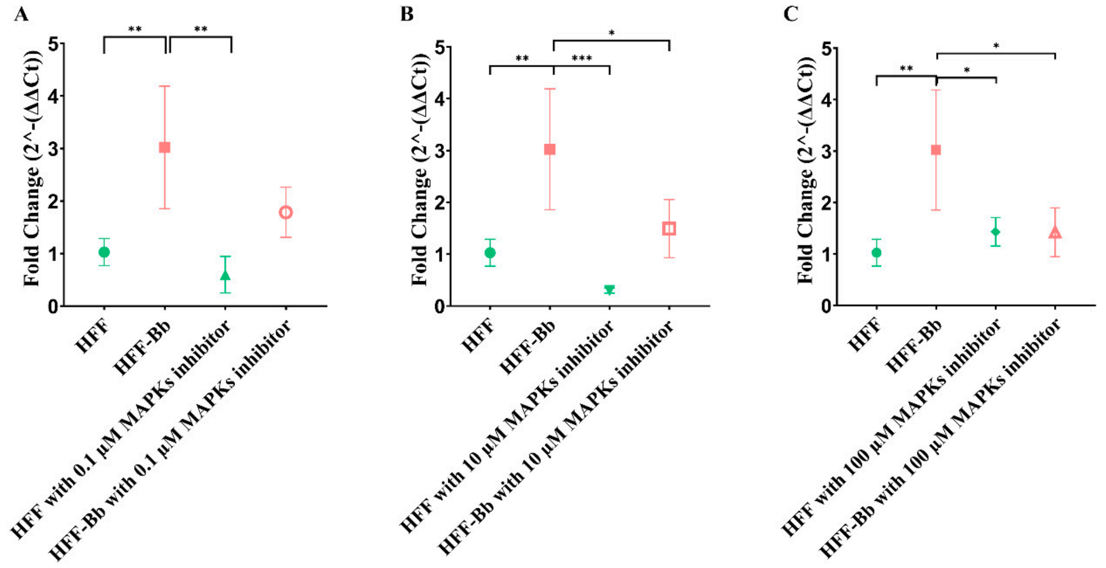

In vitro assay carried out in HFF cells infected with B. besnoiti tachyzoites and treated with ERK inhibitor.

Materials and methods

Human foreskin fibroblasts (HFF) cells were seeded at a density of 1 × 10^5 cells per well in 6-well plates. After 48 hours, upon reaching confluency, cells were divided into four experimental groups (with each group comprising three replicate wells): 1) non-infected and non-treated cells serving as the negative control (HFF), 2) cells infected with *Besnoitia besnoiti* tachyzoites (HFF-Bb), 3) cells treated exclusively with the ERK inhibitor FR180204 (Merck) and 4) cells treated with the ERK inhibitor followed by infection with *B. besnoiti* tachyzoites. The parasite infection was conducted at a multiplicity of infection (MOI) of 10:1. For inhibitor treatment, cells were pre-incubated with FR180204 at concentrations of 0.1 µM, 10 µM, and 100 µM for 1 hour before infection or further processing. Cells were harvested at 12 and 32 h pi by scraping. Four replicates of each condition were carried out. Total RNA was extracted from the cell pellets using the RNeasy Mini Kit (Qiagen), following cell lysis with QIAshredder (Qiagen) according to the manufacturer's instructions. RNA concentration and purity were measured spectrophotometrically using a NanoPhotometer Classic (Implen, USA). In addition, RNA integrity was evaluated by 1% agarose gel electrophoresis with GelRed™ staining (Biotium). Afterwards, reverse transcription was performed using the master mix SuperScript® VILO™ cDNA Synthesis Kit (Invitrogen) in a 20 µL reaction using up to 2.5 µg of total RNA. cDNA was sequentially diluted to 1:20, 1:80, 1:320 and 1:1,280, and all dilutions were analysed by qPCR. qPCRs were performed in 25 µL volumes using 12.5 µL of Power SYBR®PCR Master Mix (Applied Biosystems, USA), 10 pmol of each primer (Forward: GCTGTAATGACGAAAGTCTG; reverse: GGAAGCTCATCTCTCCTATG; Lagrée et al., 2020) and 5 µL of the diluted cDNA samples. Reactions were performed in an ABI 7500 FAST Real Time PCR System (Applied

Biosystems). Relative expression was calculated using the comparative method $2^{-\Delta\Delta Ct}$ (Livak and Schmittgen, 2001) after normalization with the housekeeping gene β-actin (Puech et al., 2015, Horcajo et al., 2017). For statistical analysis, Tukey's multiple comparison test was conducted to detect potential significant differences between means + SD from non-infected HFF (HFF), *B. besnoiti* infected HFF (HFF-Bb), HFF treated with MAPKs inhibitor and HFF-Bb treated with MAPKs inhibitor. Each dose of MAPKs inhibitor (0.1 μM, 10 μM, and 100 μM) was analysed separately.

Results

Higher *VEGFA* expression levels corresponded to HFF-Bb that decreased when they were treated with the inhibitor regardless the drug concentration. Statistically significant differences were found between HFF-Bb and HFF (p-value <0.01). In addition, *VEGFA* expression levels were higher in HFF-Bb compared to HFF-Bb treated 10 μM and 100 μM MAPKs inhibitor (p-value <0.05).

References

Lagrée, A.; Fasani, F.; Rouxel, C.; Pivet, M.; Pourcelot, M.; Fablet, A.; Romey, A.; Caignard, G.; Vitour, D.; Blaise-Boisseau, S.; Kiéda, C.; Boulouis, H.; Haddad, N.; Grillon, C. Bovine organospecific microvascular endothelial cell lines as new and relevant in vitro models to study viral infections. *Int J Mol Sci* **2020**, *21(15)*, 5249. https://doi.org/10.3390/ijms21155249

Livak, K.J.; Schmittgen, T.D. Analysis of relative gene expression data using real-time quantitative PCR and the 2(-Delta Delta C(T)) Method. *Methods* **2001**, *25(4)*, 402–408. https://doi.org/10.1006/meth.2001.1262

Puech, C.; Dedieu, L.; Chantal, I.; Rodrigues, V. Design and evaluation of a unique SYBR green real-time RT-PCR assay for quantification of five major cytokines in cattle, sheep and goats. *BMC Vet Res* **2015**, *11(1)*, 65. https://doi.org/10.1186/s12917-015-0382-0

Horcajo, P.; Jiménez-Pelayo, L.; García-Sánchez, M.; Regidor-Cerrillo, J.; Collantes-Fernández, E.; Rozas, D.; Hambruch, N.; Pfarrer, C.; Ortega-Mora, L.M. Transcriptome modulation of bovine trophoblast cells in vitro by *Neospora caninum*. *Int J Parasitol* **2017**, *47(12)*, 791–799. https://doi.org/10.1016/j.ijpara.2017.08.007
