# Supplementary material for: Transcriptomics of Besnoitia besnoiti-Infected Fibroblasts Reveals Hallmarks of Early Fibrosis and Cancer Progression
Source: Microorganisms. 2024 Mar 15;12(3):586. doi: 10.3390/microorganisms12030586 (PMC10975890; doi:10.3390/microorganisms12030586)
Supplement: Supplementary file 1 [file microorganisms-12-00586-s001.zip › Supplementary Table S1.pdf]

Supplementary Table S1. Sequences of primers used for quantitative real-time PCR (RT–qPCR) for *Bos taurus* genes.

Target	Primer sequence	References
MMP9	F: 5’ TCGACGTGAAGACACAGAAGGT 3’<br>R: 5’ TGATCCTGGCAGAAGTAAGCTTTC 3’	Kizaki et al., 2008
MMP16	F: 5’ ACCCCAGGATGTCAGTGC 3’<br>R: 5’ AATAGCTTTACGGGTTTCAGG 3’	Milner et al., 2006
PLAUR	F: 5’ ACCACACCTTCCACTTCCTG 3’<br>R: 5’ CTGGGTGGTTACAGCCACTT 3’	García et al., 2014
FGF1	F: 5’ GCTGAAGGAGAAACCACGAC 3’<br>R: 5’ GTTTTCCTCCAACCTTTCCA 3’	Berisha et al., 2006
TGFβ1	F: 5’ TGAGCCAGAGGCGGACTACT 3’<br>R: 5’ TGCCGTATTCCACCATTAGCA 3’	Sugawara et al., et al., 2010
AREG	F: 5’ CTATAGCTGCTTTCGTCTCTGC 3’<br>R: 5’ CGTTCTTCAGCGACACCTTCA 3’	Shrestha et al., 2015
FosB	F: 5’ GAAGTGTAGGAACCGGCGAA 3’<br>R: 5’ TTCTCCTTTTGGAGCTCGGC 3’	Fernández-Álvarez et al., 2023
β-actin	F: 5’ACACCGCAACCAGTTCGCCAT 3’<br>R: 5’ GTCAGGATGCCTCTCTTGCT 3’	Horcajo et al., 2017

References

Kizaki K.; Ushizawa K.; Takahashi T.; Yamada O.; Todoroki J.; Sato T.; Ito A.; Hashizume K. Gelatinase (MMP-2 and -9) expression profiles during gestation in the bovine endometrium. *Reprod Biol Endocrinol* 2008, *6*, 66. https://doi.org/10.1186/1477-7827-6-66

Milner J.M.; Rowan A.D.; Cawston T.E.; Young D.A. Metalloproteinase and inhibitor expression profiling of resorbing carti-lage reveals pro-collagenase activation as a critical step for collagenolysis. *Arthritis Res Ther* 2006, *8(5)*, R142. https://doi.org/10.1186/ar2034

García D.C.; Miceli D.C.; Valdecantos P.A.; García E.V.; Roldán-Olarte M. Expression of urokinase type plasminogen activator receptor (uPAR) in the bovine oviduct: Relationship with uPA effect on oviductal epithelial cells. *Res Vet Sci* 2014, *97(1),* 118–123. https://doi.org/10.1016/j.rvsc.2014.05.012

Berisha B.; Welter H.; Shimizu T.; Miyamoto A.; Meyer H.H.; Schams D. Expression of fibroblast growth factor 1 (FGF1) and FGF7 in mature follicles during the periovulatory period after GnRH in the cow. *J Reprod Dev* 2006, *52(2),* 307–313. https://doi.org/10.1262/jrd.17077

Sugawara K.; Kizaki K.; Herath C.B.; Hasegawa Y.; Hashizume K. Transforming growth factor beta family expression at the bovine feto-maternal interface. *Reprod Biol Endocrinol* 2010, *8,* 120. https://doi.org/10.1186/1477-7827-8-120

Shrestha K.; Lukasik K.; Baufeld A.; Vanselow J.; Moallem U.; Meidan R. Regulation of ovulatory genes in bovine granulosa cells: lessons from siRNA silencing of PTGS2. *Reproduction* 2015, *149(1),* 21–29. https://doi.org/10.1530/REP-14-0337

Fernández-Álvarez, M.; Horcajo, P.; Jiménez-Meléndez, A.; Diezma-Díaz, C.; Ferre, I.; Pastor-Fernández, I.; Ortega-Mora, L.M.; Álvarez-García, G. Transcriptional changes associated with apoptosis and type I IFN underlie the early interaction be-tween *Besnoitia Besnoiti* tachyzoites and monocyte-derived macrophages. *Int J Parasitol* 2023, *53(9),* 505–521. https://doi.org/10.1016/j.ijpara.2023.05.002

Horcajo, P.; Jiménez-Pelayo, L.; García-Sánchez, M.; Regidor-Cerrillo, J.; Collantes-Fernández, E.; Rozas, D.; Hambruch, N.; Pfarrer, C.; Ortega-Mora, L.M. Transcriptome modulation of bovine trophoblast cells in vitro by *Neospora caninum*. *Int J Parasitol* 2017, *47(12),* 791–799. https://doi.org/10.1016/j.ijpara.2017.08.007
