# Supplementary material for: Transcriptomics of Besnoitia besnoiti-Infected Fibroblasts Reveals Hallmarks of Early Fibrosis and Cancer Progression
Source: Microorganisms. 2024 Mar 15;12(3):586. doi: 10.3390/microorganisms12030586 (PMC10975890; doi:10.3390/microorganisms12030586)
Supplement: Supplementary file 1 [file microorganisms-12-00586-s001.zip › Supplementary Table S2.pdf]

Supplementary Table S2. RNA-Seq overview and mapping.

FI-Bb, Fibroblasts infected with *Besnoitia besnoiti* tachyzoites; FI, non-infected Fibroblasts.

	Samples	Nº initial reads	Nº mapped reads	% mapped reads	Nº *Bos taurus* mapped reads	% *Bos taurus* mapped reads
**Infected 12 h p.i.**	FI-Bb R1	65.874.627	43.255.868	65.66	42.907.502	65.14
	FI-Bb R2	47.529.525	36.334.128	76.45	35.486.610	74.66
	FI-Bb R3	56.335.028	43.934.064	77.99	43.416.724	77.07
**Infected 32.h p.i.**	FI-Bb R1	81.012.164	55.562.260	68.59	53.939.936	66.58
	FI-Bb R2	45.851.628	33.969.166	74.08	30.469.456	66.45
	FI-Bb R3	47.688.895	34.535.722	72.42	32.565.608	68.29
**Non-infected**	FI R1	51.300.764	38.976.506	75.98	38.976.490	75.98
	FI R2	46.001.179	35.612.846	77.42	35.612.836	77.42
	FI R3	52.723.448	40.006.340	75.88	40.006.298	75.88
