# Supplementary figures and images for: Transcriptomics of Besnoitia besnoiti-Infected Fibroblasts Reveals Hallmarks of Early Fibrosis and Cancer Progression
Source: Microorganisms. 2024 Mar 15;12(3):586. doi: 10.3390/microorganisms12030586 (PMC10975890; doi:10.3390/microorganisms12030586)

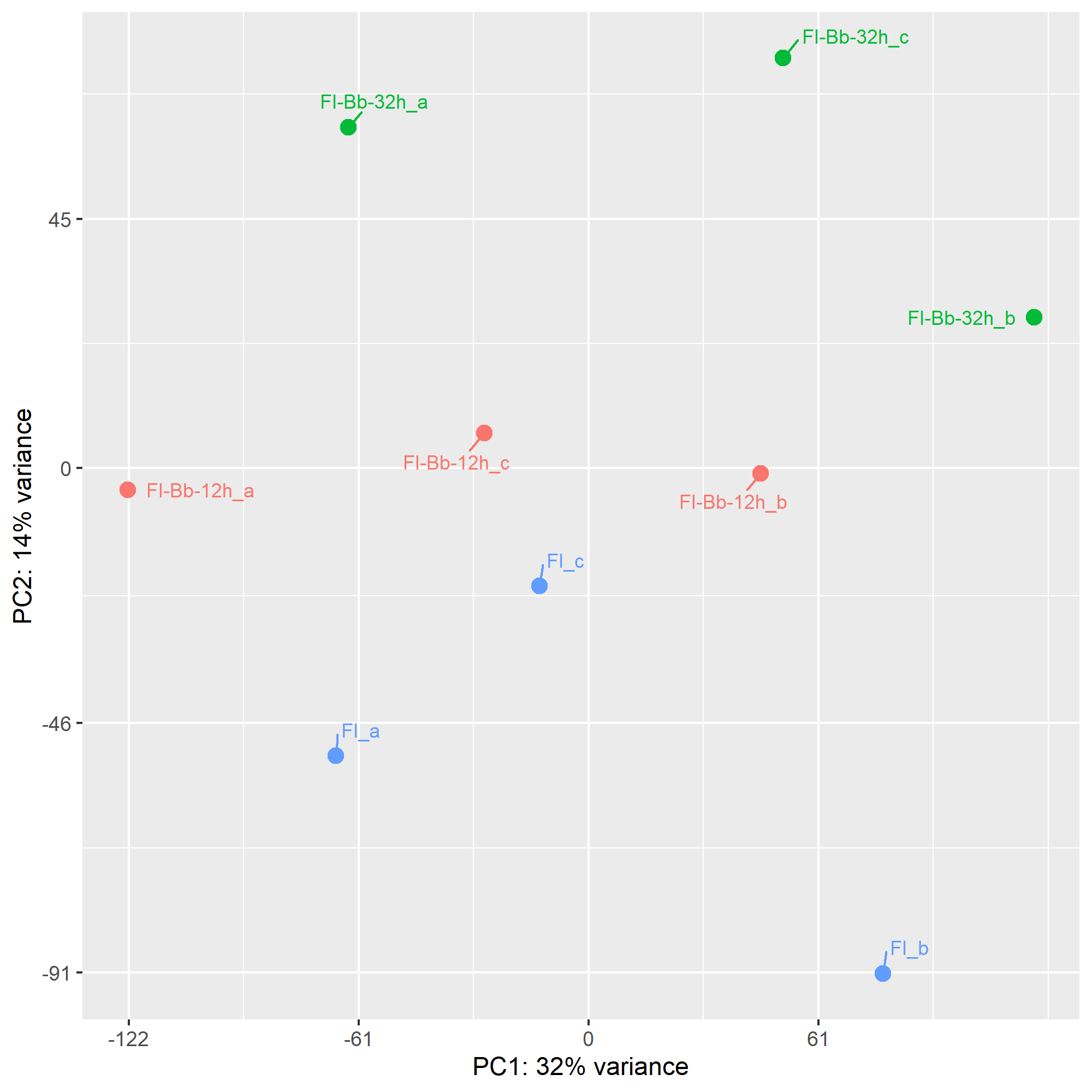

Supplement: Supplementary file 1 [file microorganisms-12-00586-s001.zip › Supplementary Figure S1.png]
